# The association between naturalistic use of psychedelics and co-occurring substance use disorders

**DOI:** 10.3389/fpsyt.2022.1066369

**Published:** 2023-01-10

**Authors:** Jonina Rabinowitz, Shaul Lev-Ran, Raz Gross

**Affiliations:** ^1^School of Public Health, Sackler Faculty of Medicine, Tel Aviv University, Tel Aviv-Yafo, Israel; ^2^Lev Hasharon Medical Center, Netanya, Israel; ^3^Sackler Faculty of Medicine, Tel Aviv University, Tel Aviv-Yafo, Israel; ^4^Israel Center on Addictions, Netanya, Israel; ^5^Division of Psychiatry, Sheba Medical Center, Ramat Gan, Israel

**Keywords:** psychedelic agents, mescaline, addiction, substance use disorder, population study

## Abstract

**Objective:**

Classic psychedelics (LSD, psilocybin, and peyote/mescaline) have been used to support addiction treatment in a variety of contexts ranging from ceremonial use to clinical trials. The aim of this study was to test the hypothesis that past naturalistic use of classic psychedelics would be associated with decreased prevalence of substance use disorder, when controlling for known confounders.

**Methods:**

This cross-sectional study used 2017 NSDUH survey data to evaluate the association between past use of the classic psychedelics LSD, psilocybin and peyote/mescaline and past year substance dependence or abuse. We calculated adjusted odds ratios by multivariate logistic regression, controlling for a range of sociodemographic variables, use of non-psychedelic illicit drugs and mental health related variables.

**Results:**

A total of 56,276 participants were included in this study. Past use of LSD and psilocybin were associated with increased odds of substance dependence or abuse compared to those who had never used psychedelics before, and this was more likely for those who had used LSD more recently. However, prior use of peyote or mescaline was associated with lower odds of past year substance dependence or abuse compared to people who had never used psychedelics before (aOR = 0.68, *p* < 0.001). Past use of classic psychedelics was not associated with nicotine dependence.

**Conclusion:**

Past use of peyote/mescaline was associated with decreased odds of substance use disorder compared to people who had never used psychedelics before, while past use of LSD or psilocybin was not. It remains unclear whether this difference is due to pharmacological differences between these compounds or simply due to the context in which peyote/mescaline are traditionally taken. Future research should investigate why naturalistic use of different psychedelics is associated with different substance use disorder effects.

## 1. Introduction

Alcohol and drug addiction pose a major threat to public health (1). In 2010, illicit drug dependence accounted for 20 million disability adjusted life years (2). In 2015, alcohol and tobacco use cost the human population over a quarter billion disability adjusted life years and illicit drug use cost an additional tens of millions of disability adjusted life years (3, 4). The public health burden of these diseases is likely to grow as the global prevalence of substance use disorders continues to rise (4).

Substance use disorders (SUD) are complex conditions and treatment is not effective for many patients (5). Relapse rates for addictions are similar to those of chronic diseases like diabetes and asthma (6). Current treatment for SUD is multifaceted, with treatment outcomes dependent on the extent and nature of the patient’s problems, appropriateness of treatment used to address those problems, and the quality of interaction between the patient and treatment providers (5, 6).

In the first wave of psychedelic research in the 1950’s, LSD was investigated in the treatment of alcohol dependence (7). A recent meta-analysis of six randomized controlled trials from this period showed that a “single dose of LSD, in the context of various alcoholism treatment programs, is associated with a decrease in alcohol misuse” (8). In recent years there has been renewed interest in the use of classic psychedelics to aid in the treatment of SUD (9, 10).

Classic psychedelics are serotonin 2A (5-HT_2A_) receptor agonists, such as lysergic acid diethylamide (LSD), psilocybin and mescaline that induce hallucinogenic and mystical-type experiences and have anti-depressive, anxiolytic, and antiaddictive effects (10–13). Though the exact anti-addictive mechanism of action is unknown, there is substantial evidence that drugs of abuse modulate serotonin transmission in the 5-HT raphe nuclei and their forebrain projections. Furthermore, 5-HT receptors seem to play a role in impulsivity, a behavioral characteristic that contributes to an individual’s vulnerability to addiction (14). As such, effective pharmacological manipulation of the 5-HT system may be the mechanism which is contributing to successful recovery from the repeating cycle of addiction by alleviating some of the neurochemical abnormalities associated with drugs of abuse (14).

The 5-HT_2A_ receptor agonism in frontal and limbic areas of the brain by classic psychedelics increase glutamatergic transmission and neuroplasticity (12), so much so that research has shown that just one or two doses can have enduring positive outcomes for individuals with SUD (10). Classic psychedelics both enable reorganization of disordered neural pathways in the default mode network and attenuate maladaptive signaling in the mesolimbic reward circuitry that plays a central role in addiction (10). Persistent changes in substance use behavior seen after psychedelic experiences may be due to these neurological processes, but also seem to be mediated by psychological responses to the highly meaningful and mystical experience from psychedelics (9), similar to brain changes seen after traumatic events (15). Such profound mystical experiences are associated with sudden and lasting behavioral change, such as long-term abstinence from alcohol (7, 15–17).

Still, the plasticity-inducing effects of 5-HT_2A_ receptor agonism make the psychedelic experience especially sensitive to context (18). This context is commonly referred to as “set and setting” (15, 19, 20). Set and setting theory proposes that psychedelics act as non-specific amplifiers of the contents of one’s consciousness (19). Therefore, one’s preparation, expectation, physical environment, and even cultural attitudes shape the effects of a psychedelic experience. Contemporary research has found that set and setting is so important for realizing therapeutic effect because psychedelics put the user in a state of enhanced suggestibility (21, 22). In fact, in studies in which set and setting were neglected, therapeutic outcomes were less positive (18).

It is for this reason that researchers believe that many indigenous groups have participated in sacramental ritual use of psychedelics for religious and pedagogical purposes since ancient times (11, 23). Examples of such sacramental use include religious consumption of ayahuasca (12, 23) and the religious sacrament performed by the Native American Church (NAC) in which peyote is consumed as part of an all-night communal prayer and song ceremony for the purpose of healing physical or spiritual imbalances that an individual or the community may be experiencing (15, 24–26). Similarly, ibogaine is often used in ceremonial contexts and in clinical research for addiction treatment (27, 28). Use in these contexts has been associated with remission of psychopathologies such as depression, anxiety, and SUD (12).

Current research continues to show the efficacy of treating various addictive disorders with psychedelic assisted psychotherapy in the clinical context (9, 29–33). In addition to the extraordinary effects of psychedelic assisted psychotherapy seen in clinical trials, large population-based studies of naturalistic (i.e., non-laboratory) use of psychedelics have shown the power of these compounds to affect mood, behavior, and even increase openness (15, 34).

One of the largest population studies on psychedelic use sought to evaluate the effect of naturalistic use of psychedelics on mental health. The results of this study showed that use of psychedelics was not only *not* an independent risk factor for mental health problems, but that psychedelic use was indeed associated with a *lower rate* of mental health problems (35). Further epidemiological studies have suggested that naturalistic use of classic psychedelics is associated with positive mental health outcomes (11, 34–37), including smoking cessation (38) and decreased risk of opioid use disorder (39, 40). These studies of naturalistic use of psychedelics are particularly important for our understanding of psychedelics because of the central role played by set and setting in mediating therapeutic effects and because naturalistic psychedelic use happens outside of controlled settings.

While past epidemiological studies have shown significant association between past use of classic psychedelics and specific substance use disorders, there are no known large population-based studies which look at the association between naturalistic use of classic psychedelics categorically across several compounds and the broad category of substance use disorders. Therefore, the goal of this study is to validate the effect of naturalistic use of classic psychedelics seen in substance use disorders across classic psychedelic compounds. This large cross-sectional study compares the prevalence of substance use disorder among individuals who have and have not been exposed to the classic psychedelics LSD, psilocybin and peyote/mescaline as a class of substances and individually. Based on past research, we hypothesized that individuals exposed to any of these classic psychedelics would have lower rates of substance use disorder when controlling for demographic variables and for confounding by co-occurring mental illness.

## 2. Materials and methods

This cross-sectional study used data from the 2017 National Survey on Drug Use and Health (NSDUH). The 2017 NSDUH survey provides estimates of substance use and mental health indicators from a representative sample of the US civilian non-institutionalized population. The data included individuals 12 years of age and older.

### 2.1. Variable selection

#### 2.1.1. Lifetime use of classic psychedelics

A new variable indicating lifetime use of classic psychedelics which was coded as “1” if the individual reported having ever used LSD, psilocybin, peyote, or mescaline. Each of the substances were also analyzed as individual substances and therefore each substance was represented with its own variable, except for peyote and mescaline which were collapsed into one variable since mescaline is the active compound in the peyote cactus.

#### 2.1.2. LSD recency

Lysergic acid diethylamide was the only classic psychedelic in the 2017 survey that included questions on recency of use. This variable was included to investigate whether there were differences in substance dependence or abuse among individuals based on how long ago they had last used LSD in comparison to individuals who had never used LSD before.

#### 2.1.3. Past year illicit drug or alcohol dependence or abuse

The DSM-4 had two diagnoses: substance dependence and substance abuse. In the DSM-5, these diagnoses were collapsed into one diagnosis–substance use disorder (mild to severe) (41). Hence, the variable “past year illicit drug or alcohol dependence or abuse” was chosen as the primary outcome variable as it captures the central components to the diagnosis of substance use disorder (SUD).

#### 2.1.4. Nicotine dependence

This variable was added as a secondary dependent variable because, unlike past year illicit drug or alcohol dependence or abuse, this variable included information on substance cravings. While the DSM-5 diagnosis of SUD largely focuses on substance dependence and abuse criteria, it also includes criteria on substance cravings. The Nicotine Dependence Syndrome Scale (or NDSS) assesses dependence on nicotine and includes criteria on nicotine craving (42). As such, nicotine dependence based on NDSS score was included as an additional dependent variable to investigate whether past exposure to classic psychedelics was differentially associated with nicotine dependence.

#### 2.1.5. Control variables

This study investigated the presence of substance dependence or abuse among individuals who have and have not been exposed to classic psychedelics. Because SUD so often co-occurs with other mental illness, (43, 44) a categorical variable on presence and severity of past year mental illness was included as a control variable. In addition, Krebs and Johansen paper (35), which was a study using NSDUH data from previous years, compared various mental health outcomes among psychedelic users and non-users and included the following control variables which were also included in this study: age at interview, sex, race/ethnicity, education, household income, marital status, likes to test self with risky behavior, and lifetime non-medical use of each of ten types of drugs: cannabis (marijuana), opiates (heroin, opiate pain relievers), cocaine, tranquilizers/sedatives (benzodiazepines, barbiturates), stimulants (amphetamine, methamphetamine, methylphenidate), MDMA (ecstasy), inhaled anesthetics (nitrous oxide, ether), alkyl nitrites (poppers), other inhalants (solvents, volatile chemicals), and PCP (phencyclidine). The Krebs and Johansen study (35) also included a control variable on lifetime exposure to an extremely stressful event. The 2017 NSDUH survey did not include any such variable and thus this control variable was not included in the study.

### 2.2. Data analysis

Regression analysis was done to find the ratio of the odds of having past year illicit drug or alcohol dependence or abuse among individuals who used classic psychedelics in their lifetime compared to those that had never used before. The binary logistic regression was first run to get the unadjusted odds ratio, and then another logistic regression was run which included control variables. The exposure variables (lifetime use of classic psychedelics) were then separated into three individual variables: lifetime use of LSD, lifetime use of psilocybin and lifetime use of peyote/mescaline and the same regression analysis was run, again using past year illicit drug or alcohol dependence or abuse as the dependent variable.

Another set of logistic regressions was run using nicotine dependence as the dependent variable, first by using the variable lifetime use of classic psychedelics as one binary variable and then by differentiating between the three categories of classic psychedelics. These regressions were also run first without and then with the control variables mentioned above. Lastly, a series of logistic regressions was run using recency of LSD use as the predictor variable in place of lifetime exposure to classic psychedelics. No power analysis was performed because the sample size was *n* = 56,276 and there were 21 variables included in the analysis, suggesting that power would be well above 90% even for detecting a small effect size (45).

All data analysis was done using SPSS version 27. Significance was set at the *p* < 0.05 level, adjusted using the Bonferroni correction when appropriate. The Bonferroni correction was applied for all regression analyses which differentiated between various psychedelics such that the adjusted significance level was considered at the *p* < 0.0167 level. All tests were two-sided.

## 3. Results

There were 56,276 individuals included in this study, 27,037 males (48%) and 29,239 females (52%). Lifetime exposure to at least one of the classic psychedelics was reported by 6,362 individuals (11.3%), 4,688 of those had been exposed to LSD at least once over the course of their lifetime (8.3%), 4,614 had been exposed to psilocybin (8.2%), and 1,285 had been exposed to either peyote or mescaline (2.3%). Further summary data can be found in Table 1.

**TABLE 1:** Summary demographic and mental health data of individuals who have and have not used any of the classic psychedelics included in this study.

		Lifetime use of classic psychedelics				Total	
		No		Yes			
		N	%	N	%	N	%
Age category, years	12–17	13433	26.9%	289	4.5%	13722	24.4%
	18–25	12144	24.3%	1696	26.7%	13840	24.6%
	26–34	7461	14.9%	1325	20.8%	8786	15.6%
	35–49	9289	18.6%	1925	30.3%	11214	19.9%
	50–64	4103	8.2%	894	14.1%	4997	8.9%
	≥65	3484	7.0%	233	3.7%	3717	6.6%
Sex	Male	23160	46.4%	3877	60.9%	27037	48.0%
	Female	26754	53.6%	2485	39.1%	29239	52.0%
Education level	Less than high school	4842	9.7%	553	8.7%	5395	9.6%
	High school grad	9763	19.6%	1506	23.7%	11269	20.0%
	Some college/Associate degree	11967	24.0%	2321	36.5%	14288	25.4%
	College graduate	9909	19.9%	1693	26.6%	11602	20.6%
	12–17 years old	13433	26.9%	289	4.5%	13722	24.4%
Race	Non-Hispanic white	28176	56.4%	4941	77.7%	33117	58.8%
	Non-Hispanic black/African American	6853	13.7%	194	3.0%	7047	12.5%
	Non-Hispanic native American/Alaskan native	722	1.4%	124	1.9%	846	1.5%
	Non-Hispanic native Hawaiian/Other Pacific Islander	244	0.5%	16	0.3%	260	0.5%
	Non-Hispanic Asian	2525	5.1%	106	1.7%	2631	4.7%
	Non-Hispanic more than one race	1854	3.7%	305	4.8%	2159	3.8%
	Hispanic	9540	19.1%	676	10.6%	10216	18.2%
Past year mental illness	None	29039	58.2%	4002	62.9%	33041	58.7%
	Mild	3578	7.2%	820	12.9%	4398	7.8%
	Moderate	1964	3.9%	555	8.7%	2519	4.5%
	Serious	1900	3.8%	696	10.9%	2596	4.6%
	Inconclusive	13433	26.9%	289	4.5%	13722	24.4%
Marital status	Married	15448	30.9%	2207	34.7%	17655	31.4%
	Widowed	1114	2.2%	85	1.3%	1199	2.1%
	Divorced or separated	3627	7.3%	840	13.2%	4467	7.9%
	Never been married	23184	46.4%	3186	50.1%	26370	46.9%
	Respondent is ≤14 years old	6541	13.1%	44	0.7%	6585	11.7%
Total family income	Less than $20,000	9391	18.8%	1185	18.6%	10576	18.8%
	$20,000–$49,999	15300	30.7%	1921	30.2%	17221	30.6%
	$50,000–$74,999	7701	15.4%	1046	16.4%	8747	15.5%
	$75,000 or more	17522	35.1%	2210	34.7%	19732	35.1%
Likes to test self with risky behavior	Unknown/refused to answer	348	0.7%	14	0.2%	362	0.6%
	Never	24333	48.7%	1590	25.0%	25923	46.1%
	Seldom	16234	32.5%	2740	43.1%	18974	33.7%
	Sometimes	7895	15.8%	1726	27.1%	9621	17.1%
	Always	1104	2.2%	292	4.6%	1396	2.5%
Lifetime non-medical use of marijuana	No	32236	64.6%	351	5.5%	32587	57.9%
	Yes	17678	35.4%	6011	94.5%	23689	42.1%
Lifetime non-medical use of heroin or opiate pain relievers	No/Unknown	46375	92.9%	3628	57.0%	50003	88.9%
	Yes	3539	7.1%	2734	43.0%	6273	11.1%
Lifetime use of cocaine	No/Unknown	47232	94.6%	2296	36.1%	49528	88.0%
	Yes	2682	5.4%	4066	63.9%	6748	12.0%
Lifetime non-medical use of tranquilizers or sedatives	No/Unknown	48465	97.1%	4742	74.5%	53207	94.5%
	Yes	1449	2.9%	1620	25.5%	3069	5.5%
Lifetime non-medical use of stimulants	No/Unknown	48537	97.2%	4765	74.9%	53302	94.7%
	Yes	1377	2.8%	1597	25.1%	2974	5.3%
Lifetime use of ecstasy (MDMA)	No/Unknown	48549	97.3%	3354	52.7%	51903	92.2%
	Yes	1365	2.7%	3008	47.3%	4373	7.8%
Lifetime non-medical use of inhaled anesthetics (nitrous oxide, ether)	No/Unknown	49221	98.6%	4601	72.3%	53822	95.6%
	Yes	693	1.4%	1761	27.7%	2454	4.4%
Lifetime use of inhaled amyl nitrite, “poppers,” rush, etc.	No/Unknown	49517	99.2%	5676	89.2%	55193	98.1%
	Yes	397	0.8%	686	10.8%	1083	1.9%
Lifetime non-medical use of other inhalants	No/Unknown	46695	93.6%	3945	62.0%	50640	90.0%
	Yes	3219	6.4%	2417	38.0%	5636	10.0%
Lifetime use of PCP	No/Unknown	49772	99.7%	5681	89.3%	55453	98.5%
	Yes	142	0.3%	681	10.7%	823	1.5%
Total		49914	100.0%	6362	100.0%	56276	100.0%

When analyzing these data to see how use of psychedelics was associated with SUD without taking into account control variables, we saw an unadjusted OR = 6.03 (*p* < 0.001), indicating that lifetime exposure to classic psychedelics was associated with six times greater odds of past year illicit drug or alcohol dependence or abuse. This OR was considerably attenuated after adjusting for the above-mentioned control variables (aOR = 1.21, *p* < 0.001).

The unadjusted odds of past year drug or alcohol dependence or abuse among individuals exposed to LSD in their lifetimes was 5.73 (*p* < 0.001) times greater than among those who had never used LSD. When including control variables, however, we computed an aOR of 1.41 (*p* = 0.024). This result was not considered statistically significant considering the Bonferroni corrected significance level *p* < 0.0167. Similarly, the unadjusted OR = 6.02 (*p* < 0.001) for illicit drug or alcohol dependence or abuse among those with lifetime exposure to psilocybin substantially decreased when control variables were included in the model (aOR = 1.135, *p* = 0.031).

While the unadjusted analysis for illicit drug or alcohol dependence or abuse among individuals who had been exposed to peyote or mescaline in their lifetime showed a positive association (OR = 2.97, *p* < 0.001), the adjusted model suggests that lifetime exposure to peyote or mescaline was significantly associated with *lower odds* of illicit drug or alcohol dependence or abuse in the past year (aOR = 0.68, *p* < 0.001). The results from these logistic regressions, including the attenuating affect of control variables are summarized in Table 2.

**TABLE 2:** Results of logistic regression showing adjusted odds ratio for past year substance use disorder among users of any of the classic psychedelics in this study, as a class of substances and individually.

		aOR (95% C.I.)			
		Ever used classic psychedelics	Ever used LSD	Ever used psilocybin	Ever used peyote/Mescaline
		1.21*** (1.10, 1.34)	1.141* (1.02, 1.28)	1.135* (1.01, 1.27)	0.681*** (0.57, 0.81)
Age category, years	12–17	1.00 (ref)	1.00 (ref)		
	18–25	1.231* (1.06, 1.43)	1.228* (1.06, 1.43)		
	26–34	0.956 (0.81, 1.13)	0.958 (0.81, 1.14)		
	35–49	0.871 (0.73, 1.04)	0.878 (0.73, 1.05)		
	50–64	0.679*** (0.55, 0.84)	0.713** (0.58, 0.88)		
	≥65	0.638** (0.49, 0.84)	0.672* (0.51, 089)		
Sex	Female	0.690*** (0.64, 0.74)	0.686*** (0.64, 0.74)		
Education level	Less than high school	1.00 (ref)	1.00 (ref)		
	High school grad	0.904 (0.80, 1.02)	0.904 (0.80, 1.02)		
	Some college/Associate degree	0.860* (0.76, 0.97)	0.863* (0.77, 0.97)		
	College graduate	0.939 (0.82, 1.07)	0.940 (0.82, 1.08)		
Race	Non-Hispanic white	1.00 (ref)	1.00 (ref)		
	Non-Hispanic black/African American	1.460*** (1.31, 1.63)	1.450*** (1.30, 1.62)		
	Non-Hispanic native American/Alaskan native	2.084*** (1.68, 2.59)	2.140*** (1.72, 2.66)		
	Non-Hispanic Hawaiian Native/Other Pacific Islander	1.077 (0.63, 1.84)	1.088 (0.64, 1.85)		
	Non-Hispanic Asian	1.11 (0.91, 1.35)	1.106 (0.91, 1.35)		
	Non-Hispanic more than one race	1.092 (0.93, 1.28)	1.102 (0.94, 1.30)		
	Hispanic	1.244*** (1.13, 1.37)	1.243*** (1.13, 1.37)		
Past year mental illness	None	1.00 (ref)	1.00 (ref)		
	Mild	2.049*** (1.85, 2.27)	2.047*** (1.85, 2.27)		
	Moderate	2.459*** (2.18, 2.77)	2.466*** (2.19, 2.78)		
	Severe	3.023*** (2.70, 3.39)	3.028*** (2.70, 3.39)		
Marital status	Married	1.00 (ref)	1.00 (ref)		
	Widowed	1.247 (0.89, 1.74)	1.236 (0.89, 1.72)		
	Divorced or separated	1.292*** (1.13, 1.48)	1.29*** (1.13, 1.48)		
	Never been married	1.626*** (1.47, 1.80)	1.628*** (1.47, 1.80)		
	Respondent is ≤14 years old	0.567*** (0.44, 0.726)	0.568*** (0.44, 0.73)		
Total family income	Less than $20,000	1.00 (ref)	1.00 (ref)		
	$20,000–$49,999	0.872** (0.80, 0.96)	0.871** (0.79, 0.96)		
	$50,000–$74,999	0.822** (0.73, 0.92)	0.818** (0.73, 0.92)		
	$75,000 or more	0.916 (0.83, 1.01)	0.913 (0.83, 1.01)		
Likes to test self with risky behavior	Always	1.00 (ref)	1.00 (ref)		
	Sometimes	0.261*** (0.14, 0.49)	0.259*** (0.14, 0.49)		
	Seldom	0.260*** (0.22, 0.31)	0.259*** (0.22, 0.30)		
	Never	0.384*** (0.33, 0.45)	0.382*** (0.33, 0.45)		
	Unknown/refused to answer	0.630*** (0.54, 0.73)	0.629*** (0.54, 0.73)		
Lifetime non-medical use of other drugs	Marijuana/Cannabis	3.800*** (3.47, 4.16)	3.816*** (3.49, 4.17)		
	Opiates (heroin or opiate pain relievers)	2.048*** (1.88, 2.24)	2.059*** (1.89, 2.25)		
	Cocaine	1.595*** (1.45, 1.76)	1.620*** (1.47, 1.79)		
	Tranquilizers or sedatives	1.536*** (1.38, 1.71)	1.538*** (1.38, 1.71)		
	Stimulants	1.797*** (1.62, 1.99)	1.792*** (1.62, 1.99)		
	Ecstasy/MDMA	1.132* (1.02, 1.26)	1.131* (1.02, 1.26)		
	Inhalants	1.600*** (1.43, 1.80)	1.603*** (1.43, 1.80)		
	PCP	0.884 (0.73, 1.07)	0.967 (0.80, 1.18)		
	Inhaled anesthetics (nitrous oxide, ether)	0.682*** (0.59, 0.79)	0.690*** (0.59, 0.80)		
	Amyl nitrite, “poppers,” rush, etc.	0.991 (0.83, 1.178)	1.007 (0.85, 1.20)		

*p < 0.05; **p < 0.005; ***p < 0.001.

When looking at nicotine dependence as the outcome variable, the odds ratio for individuals exposed to any psychedelic was 4.76, *p* < 0.001. When differentiating between psychedelics, OR_LSD_ = 4.97, OR_psilocybin_ = 4.42 and OR_peyote/mescaline_ = 3.67 and all were significant at the *p* < 0.001 level. When including control variables, we found no significant association between nicotine dependence and exposure to psychedelics. However, aOR_LSD_ = 1.17 (*p* = 0.0168) and aOR_peyote/mescaline_ = 0.80 (*p* = 0.018) are suggestive of a trend that might be worth investigating in future studies, albeit not statistically significant when Bonferroni correction was applied. These results are summarized in Table 3.

**TABLE 3:** Odds ratios (OR) and adjusted odds ratios (aOR) of past year illicit drug or alcohol dependence or abuse and nicotine dependence among individuals exposed to classic psychedelics in comparison to individuals in the sample who have never used these psychedelics.

	Illicit drug or alcohol dependence or abuse				Nicotine dependence			
	OR (95% CI)	*P*-value	aOR (95% CI)	*P*-value	OR (95% CI)	*P*-value	aOR (95% CI)	*P*-value
Lifetime exposure to classic psychedelics	**6.03** (5.65–6.43)	<0.001	**1.21** (1.10–1.34)	<0.001	**4.76** (4.42–5.13)	<0.001	1.09 (0.98–1.22)	0.12
Lifetime exposure to LSD	**5.73** (5.34–6.15)	<0.001	1.14 (1.02–1.28)	0.024	**4.97** (4.59–5.38)	<0.001	1.17 (1.03–1.33)	0.017
Lifetime exposure to psilocybin (mushrooms)	**6.02** (5.60–6.46)	<0.001	1.14 (1.01–1.27)	0.031	**4.42** (4.07–4.80)	<0.001	1.00 (0.88–1.14)	0.99
Lifetime exposure to peyote/mescaline	**2.97** (2.59–3.40)	<0.001	**0.68** (0.57–0.81)	<0.001	**3.67** (3.19–4.23)	<0.001	0.80 (0.67–0.96)	0.018

Bold results are significant.

When differentiating the effect on substance use disorder by recency of LSD use, participants who were exposed to LSD within the past 30 days had 18.34 greater odds of past year illicit drug or alcohol dependence or abuse than those who had never used LSD, without controlling for other variables. Adjusting for control variables, aOR_past 30 days_ = 1.972 for illicit drug or alcohol dependence or abuse. These same individuals had 4.35 times greater odds of nicotine dependence than people who had never used LSD, without controlling for other variables. All of these results were found at the *p* < 0.001 level.

Similarly, for individuals who had used LSD more than a month prior but in the past year, the odds of past year SUD were 15.65 times greater than those who had never used LSD, without controlling for other variables. When adjusting for control variables, aOR = 2.17. This group (those who had used LSD within the past year but more than 30 days prior) had 3.57 times greater odds of nicotine dependence than those who had never use LSD before, without including control variables. All these results were significant at the *p* < 0.001 level. In the absence of control variables, individuals who had last used LSD more than a year prior had 4.47 times greater odds of past year drug or alcohol dependence or abuse than those who had never used LSD before. This same group had 5.23 times greater odds of nicotine dependence without factoring in control variables. These were both found to be significant at the *p* < 0.001 level. When including control variables in the analysis, individuals whose last use of LSD was more than a year prior had just 1.14 times greater odds of nicotine dependence than those who had never used LSD before, *p* < 0.05. The adjusted odds ratio of drug or alcohol dependence for those who last used LSD more than a year prior was 0.94, though these results were not significant.

With the presence of control variables, LSD recency within the past year was significantly associated with greater odds of past year illicit drug or alcohol dependence or abuse (OR_past 30 days_ = 1.97, *p* < 0.001; OR_past 12 months_ = 2.17, *p* < 0.001). In addition, the odds of nicotine dependence were slightly greater among people who had used LSD more than a year prior to survey, compared to people who had never used LSD before (OR = 1.14, *p* < 0.05). No other associations were significant when controlling for other variables. These results are summarized in Table 4.

**TABLE 4:** Odds ratios (OR) and adjusted odds ratios (aOR) of past year illicit drug or alcohol dependence or abuse and nicotine dependence among individuals based on recency of last LSD use.

	Illicit drug or alcohol dependence or abuse				Nicotine dependence			
	OR (95% CI)	*P*-value	aOR (95% CI)	*P*-value	OR (95% CI)	*P*-value	aOR (95% CI)	*P*-value
Never used LSD		<0.001		<0.001		<0.001		0.20
Last LSD use within the past 30 days	**18.34** (13.71–24.53)	<0.001	**1.97** (1.40–2.77)	<0.001	**4.35** (3.02–6.26)	<0.001	1.13 (0.74–1.71)	0.57
Last LSD use more than 30 days but within the past 12 months	**15.65** (13.27–18.46)	<0.001	**2.17** (1.78–2.64)	<0.001	**3.57** (2.86–4.45)	<0.001	1.08 (0.83–1.39)	0.58
Last LSD use more than 12 months ago	**4.47** (4.13–4.84)	<0.001	0.94 (0.84–1.05)	0.28	**5.23** (4.80–5.69)	<0.001	**1.14** (1.01–1.29)	<0.05

Results in bold are considered significant.

## 4. Discussion

The purpose of this study was to investigate whether individuals who had used classic psychedelics in a naturalistic setting were less likely to have substance use disorder compared to those who had never used classic psychedelics. This study used a large sample of individuals who reported on their use of psychedelics, illicit drugs, alcohol, and nicotine, thereby illuminating several interesting facets of psychedelic use and its associated effects in a representative sample of the US population.

Without adjusting for control variables, the part of the population that had used psychedelics in their lifetime were indeed significantly and dramatically more likely to have substance dependence or abuse in the past year. This makes sense when considering the growing prevalence of psychedelic use among users of other drugs (26, 46, 47). Though prior psychedelic use was found to be associated with greater prevalence of substance dependence or abuse in the past year, adjusted analysis revealed a meaningful decrease in this effect. Furthermore, while lifetime exposure to classic psychedelics in general was associated with a slightly increased likelihood of past year substance dependence or abuse, differentiating between psychedelic substances in the regression analysis showed divergent effects between the different psychedelic compounds. Specifically, people who had used LSD or psilocybin in their lifetimes had slightly greater odds of substance dependence or abuse in the past year, while individuals who had used peyote or mescaline in their lifetimes had a significantly lower likelihood of substance dependence or abuse in the past year (aOR = 0.68, *p* < 0.001). This suggests that even naturalistic use of peyote/mescaline could bear a protective effect against illicit drug or alcohol dependence or abuse and may even serve as a protective factor against nicotine dependence.

Controlling for the confounding effects of age, sex, education level, race, past year mental illness severity, marital status, family income level, how often the respondent likes to test self with risky behavior, and having ever used any of the aforementioned drugs resulted in considerably adjusted odds ratios. Interestingly, the older an individual was at the time of interview seems to have resulted in lower odds of having an SUD. While this effect was not significant across the board, this does follow a trend which shows that age likely served as a confounding variable. Similarly, sex seemed like an obvious confounder as females have significantly lower odds of SUD and are similarly a smaller percentage of psychedelic users as shown in Table 1. Controlling for race revealed that some races seemed to have a significantly greater aOR for SUD compared to the non-Hispanic white population, which justifies including it as a control variable. However, future research should be done on these differences because, for example, while the Native American population had greater odds of having SUD, there are also traditions like the Native American Church which use psychedelics in a ceremonial context for healing (24). Past year mental illness and how often one likes to test oneself with risky behavior both showed clear, significant trends which also make it likely that these variables were confounders. In addition, marital status was a significant control variable, suggesting that it too was a confounding variable. As is expected, people who had used almost all other drugs which were included as a control variable had significantly greater odds of SUD, excluding users of PCP, inhaled anesthetics and amyl nitrite. People who had used inhaled anesthetics indeed had significantly lower odds of SUD which is a phenomenon worth investigating in future research as well. Lastly, the two socioeconomic status control variables, income and education level, did not seem to show any clear, significant trend. This perhaps suggests that the effect of psychedelic use on SUD is independent of socioeconomic status.

This was a cross-sectional study and so the effect of time was necessarily taken out of the equation. Nonetheless, by differentiating LSD users by how long ago they had last used LSD, we were able to see if there were immediate and/or long-term effects. Interestingly, there was a significant downward trend in unadjusted odds for SUD as time since last use of LSD passed, although no such trend was found for nicotine dependence or when adjusting for control variables.

### 4.1. Directions for future research

The difference between the various classic psychedelics studied are most obviously explained by two possibilities which should be investigated in further research: chemical/pharmacological differences between the different compounds and differences in the set and setting in which different psychedelics are used. One notable chemical difference is that mescaline is a phenylalkylamine whereas LSD and psilocybin are indoleamines (48, 49). While it is well established that classic psychedelics share a common attribute of agonist activity at the serotonin 5HT_2A_ receptor (49, 50), there is also evidence that other receptor sites are involved in bringing about the psychopharmacological effects of classic psychedelics. One difference between phenylalkylamines and indoleamines that may be consequential in explaining the differences seen between the compounds studied in this work is that phenylalkylamines, like mescaline, are selective 5-HT_2_ receptor agonists, whereas indoleamines are non-selective for 5-HT receptors (49). While the research on this is still in its early stages, it seems that the non-addictive nature of classic psychedelics is at least partially associated with serotonin 5-HT_2C_ receptor agonism (48).

Another possible explanation for why mescaline use in our study was associated with decreased SUD while LSD and psilocybin use were not, could be that mescaline is typically used in a more supportive context than LSD or psilocybin (26, 51). While the influence of set and setting was not accounted for in this study due to survey limitations, set and setting could have contributed to the different SUD outcomes seen in the LSD or psilocybin users vs. mescaline users in this study. The use of peyote/mescaline has a long history of being used in religious and therapeutic contexts and its illicit use is less common than illicit use of cocaine or cannabis (25, 51). Furthermore, LSD and psilocybin use is associated with use of other illicit substances (52–54). While this is hardly evidence for different set and setting, the possibility that mescaline users are more likely to enjoy a therapeutic context for their mescaline use than most LSD and psilocybin users is worth investigating in future research, given the extensive evidence on contextual factors (set and setting) mediating the kinds of benefits realized by psychedelic users (18, 19, 21, 23).

It is important to note that these two possible explanations for why decreased substance dependence or abuse was observed for mescaline only and not LSD or psilocybin are not mutually exclusive. Nonetheless, future research could compare the effects of LSD, psilocybin, and mescaline use for treating SUD, using a unified set and setting protocol. This could provide greater evidence for or against the hypothesis that pharmacological differences between classic psychedelics mediate therapeutic effects for treating SUD.

Furthermore, the outcome measure for this cross-sectional study was the (adjusted) odds ratio. This measure is based on the prevalence of SUD among people who had used psychedelics and not the incidence of SUD among psychedelic users. However, a cohort study of people who have used other substances and which measures the relative risk of developing SUD among psychedelic users compared to the psychedelically naïve would show greater evidence for the idea that naturalistic use of classic psychedelics can be a protective factor against substance use disorders.

### 4.2. Limitations

There were several limitations in this study. Firstly, there was incomplete information on the outcome of substance use disorder and we therefore used two response variables–illicit drug or alcohol dependence or abuse and nicotine dependence. While illicit drug or alcohol dependence or abuse was a good variable for capturing dependence or abuse of a large variety of substances that people abuse or are dependent on, this variable did not include information on substance cravings which is a criterion that is included in the DSM-5 diagnosis of substance use disorder. While there was not a singular SUD outcome variable, significant and meaningful associations were found for both outcome variables.

Another limitation was that we were not able to include several control variables, such as set and setting, frequency, dose of psychedelics used and, unlike in Krebs and Johansen study (35), “lifetime exposure to an extremely stressful event.” These would have been helpful to control for, given that recent research has highlighted the importance of consuming a greater dose for eliciting the peak experience which enable greater therapeutic effect (15, 18), how common exposure to adverse childhood events and other lifetime stressful events are among people who are substance dependent and how much harder it is for these people to decrease their consumption (55, 56).

This study was a cross-sectional study and therefore, limited in that it was unable to show temporality or causality. Nonetheless, by using lifetime use of psychedelics as the primary exposure variable and past year drug or alcohol dependence or abuse and past year nicotine dependence as the outcome variables, much of the exposure likely preceded the outcomes. Furthermore, by using the recency of LSD use variable, a better picture emerged of how the outcome variables changed as time passed since last LSD use. Though no strong conclusions could be drawn from the inclusion of this variable, it does appear that there was a downward trend of SUD among people the more time had passed since their last LSD use which should be investigated further in future research.

### 4.3. Conclusion

The main hypothesis of this study was that naturalistic use of each of the classic psychedelics LSD, psilocybin, and peyote/mescaline would be associated with a lower likelihood of substance use disorder when controlling for demographic and mental health related variables. While this was not found for LSD or psilocybin users, it was confirmed for people who had used peyote/mescaline. This study suggests that naturalistic use of different psychedelics may have different effects and should thus be investigated and compared in future research, while taking into account other possible mediating factors.

## Data availability statement

Publicly available datasets were analyzed in this study. This data can be found here: https://www.samhsa.gov/data/release/2017-national-survey-drug-use-and-health-nsduh-releases.

## Author contributions

This work was a product of the JR’s Master’s thesis at Tel Aviv University. As such, JR performed the research, analysis, and writing of this manuscript under the supervision of her thesis advisors, SL-R and RG. SL-R and RG provided their expertise in psychiatry, reviewed and approved the statistical analysis, proceeding results, and contributed their oversight to the writing of the manuscript. In addition, SL-R advised on all matters related to addiction and substance use disorders. RG advised on all matters related to epidemiology and psychopharmacology, as well as his background in clinical use of psychedelics. All authors contributed to the article and approved the submitted version.
